# Sensory processing and adaptive behavior in Phelan-McDermid syndrome: a cross-sectional study

**DOI:** 10.1007/s00431-022-04564-y

**Published:** 2022-07-15

**Authors:** Sergio Serrada-Tejeda, María-Luz Cuadrado, Rosa Mª Martínez-Piédrola, Nuria Máximo-Bocanegra, Patricia Sánchez-Herrera-Baeza, Lucía Rocío Camacho-Montaño, Marta Pérez-de-Heredia-Torres

**Affiliations:** 1 Department of Physical Therapy, Occupational Therapy, Rehabilitation and Physical Medicine, Avenida de Atenas s/n. CP.28922, Rey Juan Carlos University, Alcorcón, Madrid, Spain; 2 Department of Neurology, Hospital Clínico San Carlos, Madrid, Spain; 3 Department of Medicine, School of Medicine, Universidad Complutense, Madrid, Spain

**Keywords:** Sensory processing, Phelan-McDermid syndrome, *SHANK3*, Autism, Adaptive behavior

## Abstract

Phelan-McDermid syndrome (PMS) is a genetic disorder caused by a mutation or deletion of the *SHANK3* gene (chromosome 22q13.3), characterized by different sensory processing anomalies. The objective of this study is to expand and provide a detailed definition of the sensory profile of patients with PMS. The secondary objective was to examine the relationship between sensory patterns and adaptive behavior. A cross-sectional study was carried out among 51 Spanish patients with a confirmed genetic diagnosis of PMS. All the participants’ parents completed the Short Sensory Profile-Spanish (SSP-S) and the *Adaptive Behavior Assessment System II* (ABAS-II). Correlational, multiple regression and hierarchical cluster analyses were performed. An atypical sensory profile was identified in almost 75% of PMS patients. Definite differences were found among scores; nonetheless, sub-threshold values were observed in tactile sensitivity, underresponsive/seeks sensation, auditory filtering, and low energy/weak sensory categories. Conceptual, social, and practical domains, as well as the General Adaptive Composite (GAC) of the ABAS-II showed extremely low scores (i.e., <70). Significant correlations were found (*p*<0.005) between SSP-S scores and the conceptual, social, practical, and GAC index of the ABAS-II, whereby higher SSP-S scores were associated with better skills and higher adaptive performance. The cluster analysis indicated that the group with the largest mutation size (7.23 Mb) showed the greatest sensory processing difficulties and very low adaptive skills.

*Conclusions*: Patients with PMS show an atypical sensory profile, which correlates with limitations in general adaptive behaviors.

**Table:** 

**What is Known:** *• PMS sensory processing difficulties were associated with a pattern of underresponsive/seeks sensation, low energy/weak, and tactile hyporeactivity.* *• Sensory processing difficulties have been associated with limitations in the development of appropriate adaptive communication and interaction behaviors.*	
**What is New:** *• Sensory definite differences associated with tactile hyperreactivity, as well as significant effects of underresponsiveness/seeks sensation and auditory filtering categories on the adaptive abilities were found in SHANK3*_*deletion*_ *patients.* *• Cluster analysis suggests that smaller mutation sizes were related to better sensory processing and higher adaptive skills, while patients with larger deletion sizes have greater adaptive difficulties and worse sensory processing skills.*

**Supplementary Information:**

The online version contains supplementary material available at 10.1007/s00431-022-04564-y.

## Introduction

Clinical assessment of neurodevelopmental disorders requires the assessment of multiple aspects of development, which, due to their complexity, can delay medical diagnosis and multidisciplinary intervention [1]. This situation is common in genetic disorders, as in the case of Phelan-McDermid syndrome (PMS).

PMS is one of the most prevalent single-gene forms associated with the diagnosis of autism spectrum disorder (ASD) [2]. The use of molecular genetic tests such as chromosomal microarray analysis [3–5] as well as the identification of a characteristic clinical profile has made it possible to define the alterations observed in this genetic condition [1]. The current classification system for PMS differentiates between PMS-*SHANK3* related and PMS-*SHANK3* unrelated [6] to distinguish between those with a terminal deletion or pathogenic variants of the *SHANK3* gene [3, 7] and cases in which *SHANK3* is not involved.

The clinical signs and symptoms of PMS are variable and non-specific [8, 9], including neonatal hypotonia [8], absent or delayed language [10, 11], intellectual disability [7], and sensory abnormalities [12–15]. Given the relevance of sensory processing for the diagnosis of individuals with autistic traits, several studies have attempted to establish a predictive association between sensory phenotypes [16–21] and general maladaptive behaviors [17, 22, 23].

Although the nature of this association remains unclear, cross-sectional studies [17, 24, 25] have found that sensory hyporeactivity and sensory seeking patterns were associated with poorer socio-communicative and daily living skills, whereas sensory hyperreactivity was associated with higher communicative performance [24]. In addition, longitudinal studies [23] show that in early childhood, sensory hyporeactivity may have long-term negative implications for social outcomes, whereas hyperreactivity may predict poorer adaptive and daily living skills in later childhood [25].

In other genetic phenotypes of autism associated with severe cognitive difficulties, adaptive skills have been shown to be severely impaired [1, 26–28], and it appears that in individuals with ASD with lower cognitive skills, increased repetitive sensorimotor behaviors are observed [29, 30]. In PMS, unusual sensory responses such as exaggerated reactions to stimuli or seeking behaviors have been identified [31]. These sensory difficulties are associated with hyporeactive and low-energy profiles rather than sensory seeking patterns and lower sensory hyperreactivity in the visual, tactile, and auditory modalities (12,14-15). In addition, sensory sensitivity in PMS appears to be lower than in idiopathic ASD, suggesting that the PMS population tends to be less defensive or hyperreactive to these sensory stimuli [14].

Because previous studies have been conducted with small sample sizes and due to scarce research on PMS, the aims of the present study were twofold: (a) to expand and define in detail the sensory profile of PMS patients and (b) to examine the association between sensory patterns and adaptive behaviors. This study extends previously published work by applying cluster analysis techniques to examine sensory profile patterns and by establishing an empirical basis for understanding the relationship between sensory difficulties and adaptive behaviors.

## Methods

### Study design

A cross-sectional both descriptive and correlational design was used following the guidelines of the Strengthening the Reporting of Observational Studies in Epidemiology (STROBE) Checklist [32]. The data from this descriptive study are part of a larger longitudinal research project examining the evolution of adaptive behavior and sensory processing difficulties in the PMS population. The study was approved by the Clinical Research Ethics Committee of Universidad Rey Juan Carlos. The families of the study participants completed the informed consent document and accepted to provide supporting documentation for diagnostic confirmation.

This study was conducted in Spain, and the collection, management, storage, communication, and transfer of all data were completed in accordance with the provisions of the Declaration of Helsinki [33], the General Data Protection Regulation (EU) 2016/679 Regulation [34], and the current Spanish legislation on personal data protection [35].

### Participants

The study sample consisted of a group of 51 patients diagnosed with PMS. Convenience sampling was conducted between July and December 2020. Participants with PMS were recruited via an internal communication sent by the board of the Phelan-McDermid Syndrome Association of Spain. To participate in the study, parents signed and accepted the informed consent form.

Patients met the inclusion criteria if they had a diagnosis of PMS confirmed by demonstration of a *SHANK3* deletion with comparative genomic hybridization (CGH) or a *SHANK3* mutation demonstrated by whole exome sequencing (WES). Due to the comorbidity of the diagnosis of ASD and PMS, patients could also have a confirmed diagnosis and/or suspected features of ASD identified by a physician, psychologist, neurologist, or psychiatrist, as described in the DSM-5.

### Procedure

For the assessment of participants, a hard copy of the Spanish version of the Short Sensory Profile (SSP-S) and the *Adaptive Behavior Assessment System II* (ABAS-II) Questionnaire along with instructions was sent for their completion. Both questionnaires were completed by the primary caregiver as they are the person who most meets the child’s needs, including activities of daily living and supervising the child in an age-appropriate manner. However, when necessary, the research team assisted those who requested help in completing the questionnaire by telephone, either because of difficulties in understanding the instructions or if some of the questionnaire items remained unanswered. Specifically, three primary caregivers were contacted by telephone to resolve doubts related to the understanding of the instructions, as well as six other primary caregivers who sent documentation with several unanswered items. At no time during the telephone contact did the members of the research team provide the answers to the questionnaire; rather, they solely assisted by resolving their doubts. A separate document was also sent to collect patient-related information, including sociodemographic data and type of genetic disorder, as well as attendance to specific rehabilitation treatments.

### Variables and data measurements

Sociodemographic and genetic data were recorded for each participant, including age, gender, type of genetic alteration, place of residence, primary caregiver, and use of rehabilitation resources. Scores on the SSP-S and ABAS-II were also collected for each participant.

The Short Sensory Profile-Spanish (SSP-S) is the cross-culturally adapted and validated version of the Short Sensory Profile (SSP) for Spanish children [36, 37]. The SSP is a screening tool based on the Sensory Profile, a questionnaire designed by Dunn et al. [38] and used to identify sensory processing difficulties. It is a measure of the main report consisting of a 38-item questionnaire divided into seven sections or subscales that collect information on different sensory aspects: tactile, sensitivity, taste/olfactory sensitivity, movement sensitivity, hyporesponsiveness/sensation seeking, auditory filter, low energy/weakness, and visual/auditory sensitivity. All items are scored on a range of 1 to 5 (1, always; 2, often; 3, sometimes; 4, almost never; 5, never). Each raw score is compared with a threshold value to determine a category of performance: typical performance, probable difference (1 standard deviation below the mean), and definite difference (2 standard deviations below the mean). Lower scores indicate a higher frequency of endorsed behaviors and greater differences in sensory processing. The SSP total score and the score on each subscale can be used to classify children’s sensory profile according to the proposed categories (typical, probable difference, or definite difference), which were based on score percentiles from a large normative sample of children without disabilities (Table 1).

**Table 1:** Sociodemographic data and sensory profile score classification (SSP-S) (adapted from: Dunn [38])

**Age (years), mean (SD)**	11 (7.7)		
**Age range (years), *n* (%)**			
3–5	15 (29%)		
6–11	20 (39%)		
12–17	7 (14%)		
18–24	9 (18%)		
**Gender, *n* (%)**			
Male	25 (49%)		
Female	26 (51%)		
**Genetic alteration, *n* (%)**			
Deletion	45 (88%)		
Deletion size interval	52 kb–8.53 Mb		
Point mutation	6 (12%)		
**Main caregiver, *n* (%)**			
Mother	29 (57%)		
Father	1 (2%)		
Both parents	20 (39%)		
Another person	1 (2%)		
**Use of rehabilitation services, *n* (%)**			
Physiotherapy	24 (47%)		
Speech therapy	38 (76%)		
Psychotherapy	19 (37%)		
Occupational therapy	11 (23%)		
None	3 (6%)		
**Place of residence, n (%)**			
Spain			
Andalucía	10 (20%)		
Madrid	10 (20%)		
Valencia	5 (9.8%)		
País Vasco	3 (5.9%)		
Cataluña	5 (9.8%)		
Navarra	3 (5.9%)		
Castilla-La Mancha	3 (5.9%)		
Murcia	2 (3.9%)		
Asturias	2 (3.9%)		
Islas Baleares	2 (3.9%)		
Galicia	2 (3.9%)		
Castilla León	1 (1.9%)		
Italy	1 (1.9%)		
Argentina	2 (3.9%)		
**SSP section/subscale (items)**	**Classification**		
	Definite difference	Probable difference	Typical performance
Tactile sensitivity (1–7)	7–26	27–29	30–35
Taste/smell sensitivity (8–11)	4–11	12–14	15–20
Movement sensitivity (12–14)	3–10	11–12	13–15
Underresponsive/seeks sensation (15–21)	7–23	24–26	27–35
Auditory filtering (22–27)	6–19	20–22	23–30
Low energy/weak (28–33)	6–23	24–25	26–30
Visual/auditory sensitivity (34–38)	5–15	16–18	19–25
Total (1–38)	38–141	142–154	155–190

*SD* standard deviation, *SSP-S* Short Sensory Profile-Spanish; SSP-S’ classifications are based on the performance of children without disabilities (*n*= 1037). No missing data was reported

The *Adaptive Behavior Assessment System II* (ABAS-II) Questionnaire provides a comprehensive assessment of the adaptive skills of people from birth to 89 years of age [39]. Through the assessment of multiple environments, the ABAS-II contributes to the assessment of a person’s functional abilities and adaptive responses necessary for effective functioning. The ABAS-II assesses ten specific adaptive skill areas that are grouped into three indices or domains of adaptive behavior: the conceptual domain (communication, functional [pre-] academic skills, and self-direction), the social domain (leisure and social interaction skills), and the practical domain (use of community resources, home/school life, health and safety, self-care, motor skills, and employment). Both the adaptive skill areas and the domains are based on the definition of adaptive behavior issued by the American Association on Intellectual Developmental Disabilities (AAIDD). The ABAS-II also provides a General Adaptive Composite (GAC) that summarizes performance in all adaptive skill areas. Composite scores for conceptual, social, and practical domains, as well as the GAC, have a score of 100 and a standard deviation of 15. For each adaptive skill area, the raw scores are converted to scaled scores with a mean of 10 and a standard deviation of 3. Its psychometric properties have demonstrated high test-retest reliability (*r*>0.80) and adequate validity and internal consistency (GAC: *r*>0.90; conceptual, social, and practical indices: *r*>0.83).

### Statistical methods

Basic descriptive methods were used to describe the sample. For qualitative variables, the number of cases present in each category and the corresponding percentage were calculated. For each sample (*SHANK3*_deletion_ versus *SHANK3*_mutation_), the mean and standard deviation (SD) were calculated for quantitative variables that followed a normal distribution, and otherwise, the median and interquartile range were determined. Normality was tested with the Shapiro-Wilk test. In addition, the degree of correlation between the ABAS-II indices and the SSP-S total score was analyzed through the Spearman coefficient. To correct for type I error in multiple pairwise comparisons, multiple linear regression models were created to confirm the influence of sensory processing on the adaptive skills and domains, as well as on the GAC. For the regression models, the effect of the variables age and type of genetic alteration was also adjusted. No missing values were found for the analysis of results.

In addition, to determine the grouping of the participants, a hierarchical cluster analysis was performed on individuals with *SHANK3* deletion. Ward’s method was used, considering the following variables: sex, age, deletion size, SSP-S_total score_, and ABAS-II General Adaptive Composite (GAC).

Statistical analysis was performed with SPSS 27.0 for Windows (Copyright© 2013 IBM SPSS Corp.). Cluster analysis was carried out with the R 4.1.2 program. Statistically significant differences were those with *p* < 0.05.

## Results

### Demographic and genetic characteristics of the sample

Sixty-nine families from the Phelan-McDermid Syndrome Association of Spain were contacted (Supplementary Figure 1). The final study sample consisted of a total of 51 participants with PMS (74% response rate), 25 males and 26 females, with a mean age of 10.9 years (SD 7.7). In 45 cases (88%), the condition was associated with a deletion of the *SHANK3* gene. The size of the deleted segment was highly variable, ranging from 52 kb to 8.53 Mb. In the remaining 6 participants (11%), the genetic alteration was due to a mutation of the gene. Table 1 shows the sociodemographic and genetic data as well as the score classification for the SSP-S.

### Associations between sensory profile and adaptive behavior

Table 2 shows the descriptive analysis and frequency distribution of the SSP-S scores according to the threshold value from the cohort validated on the English version of the SSP (35). In the *SHANK3*_deletion_ sample, the mean total score, with a value of 136.5 (SD 20.14), was associated with definite differences in the sensory profile. Overall, 30 patients (59%) showed lower scores related to a definite difference (SSP_range score_= 38–141), 8 patients (16%) showed a probable difference (SSP_range score_= 142–154), and the remaining 13 participants (25%) showed typical performance scores (SSP_range score_=155–190). The subsample of *SHANK3*_mutation_ showed definite sensory processing difficulties in the same sensory subcategories as *SHANK3*_deletion_ and a mean total score, associated with probable differences in the sensory profile [143.2 (21.4)].

**Table 2:** Descriptive statistics and differential analysis of the SSP-S in PMS population (*n*=51)

**SSP-S subscales**	**Sensory profile classification**								**Independent sample tests**	
	*SHANK3*_deletion_ (*n*=45)				*SHANK3*_mutation_ (*n*=6)					
	Score	Definite difference *N* (%)	Probable difference *N* (%)	Typical performance *N* (%)	Score	Definite difference *N* (%)	Probable difference *N* (%)	Typical performance *N* (%)	Student’s *t*-test	*U*-Mann-Whitney
									*p* value^a^	*p* value^b^
Tactile sensitivity, mean (SD)	26.7 (5.0)*	25 (56%)	6 (13%)	14 (31%)	29.0 (4.2)	2 (33%)	1 (18%)	3 (59%)	0.28	---
Taste/smell sensitivity, median (IQR)	18.3 (4–20)	2 (4.4%)	2 (4.4%)	41 (91%)	20 (20-20)	6 (100%)	---	---	---	0.12
Movement sensitivity, median (IQR)	11.9 (3–15)	13 (29%)	6 (13%)	26 (58%)	12.8 (6-15)	1 (17%)	---	5 (83%)	---	0.74
Underresponsive/seeks sensation, mean (SD)	21.9 (6.8)*	27 (60%)	8 (18%)	10 (22%)	21.7 (68.8)*	4 (66.7%)	---	2 (33%)	0.93	---
Auditory filtering, mean (SD)	18.4 (5.0)*	28 (62%)	8 (18%)	9 (20%)	19.5 (4.1)*	2 (33%)	2 (33%)	2 (33%)	0.60	---
Low energy/weak, mean (SD)	19.0 (6.9)*	33 (73%)	2 (4.4%)	10 (22%)	21.3 (8.1)*	4 (67%)	---	2 (33%)	0.46	---
Visual/auditory sensitivity, median (IQR)	19.8 (9–25)	5 (11%)	8 (18%)	32 (71%)	19.0 (15-21)	1 (17%)	1 (16.7%)	4 (67%)	---	0.23
Total score, mean (SD)	135.6 (20.4)*	28 (62%)	8 (18%)	9 (20%)	143.2 (21.4)	2 (33%)		4 (67%)	0.39	---

*IQR* interquartile range, *SD* standard deviation, *SSP-S* Short Sensory Profile-Spanish

^a^Student’s *t*-test (between-groups differences if *p*<0.05)

^b^*U-*Mann-Whitney test (between-groups differences if *p*<0.05)

^*^Definite difference in sensory processing

No missing data was reported

Table 3 shows the scores obtained in the adaptive skills and domains of the ABAS-II. All the scores were indicative of extremely low performance. The analysis of differences for both groups according to genetic defect showed no statistically significant differences in either SSP or ABAS-II.

**Table 3:** Descriptive statistics and differential analysis of the ABAS-II adaptive skills and domains in PMS population (*n*=51)

**ABAS-II**	**Total sample** (*n*=51)	***SHANK3***_**deletion**_ (*n*=45)	***SHANK3***_**mutation**_ (*n*=6)	**Independent sample tests**	
				Student’s *t*-test	*U*-Mann-Whitney
				*p* value^a^	*p* value^b^
General Adaptive Composite median (IQR)	53.0 (51–53)**	54.5 (51–74)*	58.1 (51–80)**	---	0.78
Conceptual domain, median (IQR)	54.0 (53–54)**	55.1 (53–85)**	58.8 (53–78)**	---	0.16
Communication, median (IQR)	1.0 (1–1)*	1.2 (1–8)*	2.2 (1–7)*	---	0.26
Functional academics, median (IQR)	1.0 (1–1)*	1.2 (1–9)*	1.0 (1–1)*	---	0.94
Self-direction, mean (SD)	2.1 (2.5)*	1.9 (2.4)*	3.2 (3.7)*	0.28	---
Social, median (IQR)	53.0 (53–54)**	56.18 (51–94)**	67.7 (51–110)**	---	0.43
Leisure skills, mean (SD)	2.1 (2.6)*	2.0 (2.3)*	3.8 (5.2)*	0.43	---
Social interaction skills, mean (SD)	1.7 (2.2)*	1.6 (1.9)*	4.3 (4.6)*	0.21	---
Practical, median (IQR)	56.0 (52–56)**	56.9 (51–75)**	56.0 (51–69)**	---	0.58
Community use, mean (SD)	1.3 (0.9)*	1.5 (2.1)*	1.7 (1.2)*	0.90	---
Home or school living, median (IQR)	1.0 (1–5)*	2.9 (1–13)*	4.5 (1–12)*	---	0.41
Health and safety, mean (SD)	1.6 (1.5)*	1.67 (1.6)*	1.3 (0.81)*	0.62	---
Self-care, mean (SD)	1.4 (1.4)*	1.4 (1.5)*	1.2 (0.4)*	0.65	---
Motor/work, median (IQR)	1.0 (1–1)*	1.0 (1–1)*	1.0 (1–1)*	---	1.00

*IQR* interquartile range, *SD* standard deviation

^a^Student’s *t*-test (between-group differences if *p*<0.05)

^b^*U*-Mann-Whitney test (between-group differences if *p*<0.05)

^*^Atypical on ABAS-II adaptive skills subscales is ≤ 7

^**^Atypical on ABAS-II GAC/conceptual/social/practical scores is ≤ 85

No missing data was reported

The results of the correlational analysis between SSP and adaptive skills showed significant correlations (*p*<0.005) in the following sensory categories: tactile sensitivity, movement sensitivity, auditory filtering, underresponsive/seeks sensation, low energy/weak, and visual/auditory sensitivity. Because scalar motor skill scores showed no variability across patients and were associated with very low adaptive skill profiles, it was not possible to assess the degree of correlation with other variables. See Supplementary Table 1 and Scatterplot Matrix 1 for a detailed review of correlations between SSP and ABAS-II adaptive skills. Furthermore, significant correlations (*p*<0.005) between SSP-S total score and each ABAS-II domain as well as GAC index were found, whereby higher SSP-S scores were related to better skills and higher adaptive performance. See Supplementary Table 2 and Scatterplot Matrix 2 for a detailed review of correlations between SSP and ABAS-II adaptive domains.

### Linear regression models

Subsequently, a multiple linear regression model, adjusted for age and genetic defect, was performed to examine the effect of sensory categories (Supplementary Tables 3-7) as well as the SSP_total score_ (Supplementary Tables 8-9) on the ABAS-II variables that were described as significant in the previous correlational analysis (See Supplementary Table 1).

#### SSP categories and adaptive behavior

Multiple regression models that considered the sensory categories of tactile sensitivity, low energy/weak, and auditory/visual sensitivity did not show significant results. In contrast, the multiple regression analysis detected a significant effect of underresponsiveness/seeks sensation on leisure (*β*=0.10, *p*=0.043), home or school living (*β*=0.18, *p*=0.009), and health and safety adaptive skills (*β*=0.09, *p*=0.003). Similarly, the auditory filtering category showed a significant effect on the adaptive abilities of self-direction (*β*=0.20, *p*=0.005), leisure (*β*=0.22, *p*=0.004), home or school living (*β*=0.25, *p*=0.007), and health and safety (*β*=0.10, *p*=0.023), as well as on social adaptive domains (*β*=0.99, *p*=0.005) and the GAC (*β*=0.37, *p*=0.031).

Furthermore, in multiple regression models that considered the categories of movement sensitivity and auditory filtering, the genetic defect showed a significant effect on adaptive social interaction skills (*β*=−2.59, *p*=0.016; *β*=−2.61, *p*=0.015). In this case, the results indicated that participants with *SHANK3*_deletion_ show lower adaptive social interaction skills than individuals with *SHANK3*_mutation_.

#### SSP total score and adaptive behavior skills

Of the regression analyses performed that assessed the influence of SSP_total score_, only those related to the adaptive skills (Supplementary Table 8) of self-direction (*β*=0.04, *p*=0.019), leisure (*β*=0.05, *p*=0.008), and home or school living (*β*=0.06, *p*=0.007) were significant. Similarly, in the outcome model of social interaction skills, the genetic defect was significant, indicating that patients with *SHANK3*_deletion_ showed lower scores than patients with *SHANK3*_mutation_ (*β*=−2.51, *p*=0.019). On the other hand, the SSP_total score_ only showed significant effects on social (*β*=0.24, *p*=0.007) and practical domains (*β*=0.009, *p*=0.011). For the conceptual and GAC domains, no significant results were found (Supplementary Table 9).

### Exploratory cluster analysis of PMS sensory and adaptive profile heterogeneity

**Fig. 1:**
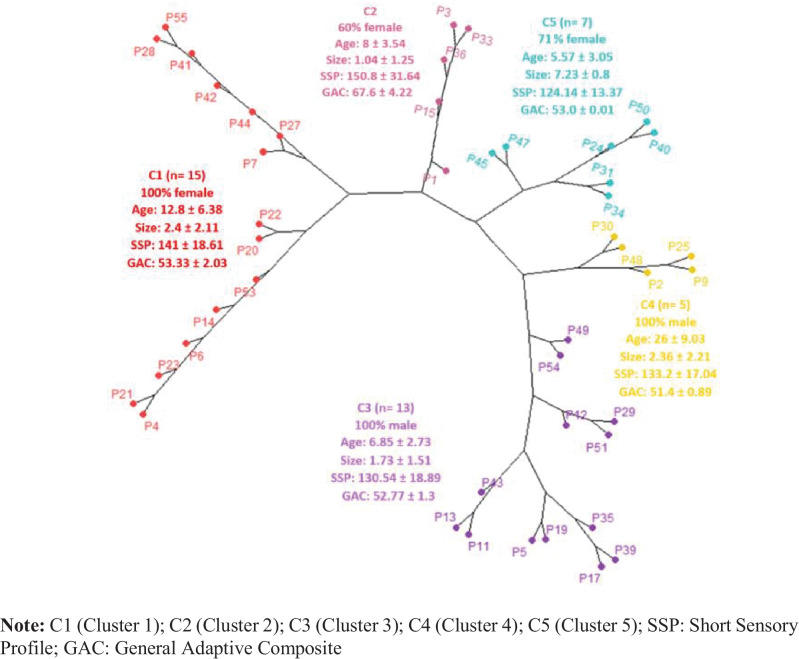
Hierarchical clustering phylogenetic dendrogram

To explore the phenotypic and genetic heterogeneity of PMS patients, a hierarchical clustering analysis was performed on 45 individuals with a *SHANK3* deletion for whom information regarding sex, age, deletion size, and SSP-S_total score_ and ABAS-II General Adaptive Composite total score was available. Considering deletion size as the main separation, five clusters summarized the variability (Fig. 1). Cluster 1 consisted only of females (*n*=15) with a medium deletion size (2.4 Mb) and scores associated with definite sensory processing dysfunction and very low adaptive skills. Cluster 2 (*n*=5) corresponded to a mixed group (3 females and 2 males) with the smallest deletion size, better scores on the sensory profile, and associated with a probable difference in sensory processing, as well as low adaptive skills. Clusters 3 and 4 (*n*=18) were both constituted by males, with a deletion size between 1.73 and 2.26 Mb, and a sensory profile associated with definite difficulties, as well as very low adaptive skills. Finally, cluster 5 (*n*=7), which was mostly constituted by females (*n*= 5), was the group with the largest deletion size (7.23Mb). This cluster showed the lowest scores of the whole sample analyzed, as well as very low adaptive behavioral skills.

## Discussion

The use of sensory assessments has been previously used in PMS patient samples [12–15]. However, this is the first study that examines the relationships between adaptive behaviors and sensory processing skills in PMS using the SSP and the ABAS-II. Moreover, it is important to explore the association between these variables, given that the presence of signs of atypical sensory reactivity is associated with difficulties in performing activities of daily living [25, 40–42] and with lower adaptive behavior and social engagement skills [17, 41].

### Sensory processing

In our study, we have identified an atypical sensory profile in patients with PMS. Almost 75% of the tested sample obtained an overall SSP-S score, consistent with a definite or probable difference compared to the general population. Considering the sensory profiles assessed, overall, the SSP score and four categories showed a clear sensory difference: unresponsiveness/sensitivity, low energy/weakness, auditory filtering, and tactile sensitivity.

In line with previous research, our study reflects that most patients with PMS (61%) show a pattern of underresponsive/seeks sensation, which may be related to high sensory thresholds and passive behavioral responses. The study by Tavasoli et al. [14], which used *The Sensory Assessment for Neurodevelopmental Disorders (SAND)*, instead of the SSP, identified percentages that reflected a high frequency of these behaviors in the PMS population. In this case, 92% of the sample showed behaviors associated with hyporesponsive patterns and 65% with sensory seeking. Similar results were also identified in previous studies employing the SPP for the assessment of patients with PMS, with definite sensory differences in underresponsive/seek sensation [12, 13, 15]. Likewise, studies on PMS by Mieses et al. [12] and Droogmans, Swillen, and Van Buggenhout [13] identified a high percentage of sensory responses associated with probable or definite differences in the low energy/weak sensory category. This alteration has been associated with vestibular and proprioceptive hyporesponsiveness and could be a distinctive feature of PMS, perhaps related to hypotonia and underlying psychomotor retardation due to genetic abnormalities.

Our study also found definite differences in 59% of the sample for the auditory filtering category (auditory filtering raw score= 19.33±7.02) and in 53% for the tactile sensitivity category (tactile sensitivity raw score= 26.94±4.97). These results are similar to those obtained by Mieses et al. [12] and Droogmans et al. [13], who identified similar raw scores and percentages in both sensory categories in the PMS sample evaluated. In the SSP, 5 of the 7 items in the tactile category were associated with sensory hyperresponsive behavior, consistent with low sensory thresholds and a high level of alertness. However, in the study by Tavasoli et al. [14], the scores obtained on the *SAND* were indicative of a tactile hyporesponsiveness profile. Considering the neural mechanisms underlying sensory abnormalities, and although the results are not comparable to our study population, animal studies [43] suggest that the atypical sensory responses of tactile hyperreactivity and somatosensory hyporeactivity may be due to deficits in central nervous system circuitry, resulting from a *SHANK3* gene deletion.

### Adaptive functioning

In terms of adaptive skills, very low levels were identified reflecting the need for generalized assistance during daily activities and routines [8, 44–46]. Recent studies that have evaluated adaptive skills in PMS [8, 11, 13, 23, 47] have used the Vineland Adaptive Behavior Scales (VABS), with similar findings, indicating very low adaptive skills (<70). Although our study is the first to use the ABAS-II in PMS population, the results obtained are similar and reflect very low levels of adaptive behavior (GAC <70) in most of the sample analyzed. In our sample, scalar scores for motor skills showed no variability across patients, although their direct scores revealed some variability. This may be because in severely disabled subjects, the ability to identify subtle differences on the ABAS-II may be limited and affected by the so-called floor effect.

In the previous literature [22, 44–46], significant associations have been identified between abilities to adequately participate in activities of daily living and atypical sensory processing, especially those related to sensory avoidance, auditory difficulties, and signs of underresponsive/sensation seeking. In fact, hyporeactive profiles have generally been associated with limitations in the development of appropriate adaptive behaviors of interaction, communication, and social participation with adults and peers [17]. In contrast, although the low energy/weak variable was also consistent with a hyporeactivity profile in the SSP and all previous literature (12-15) has highlighted significant differences in the sensory profile of the PMS in this category, our results indicate that it does not appear to significantly influence adaptive skills. Even so, in our study, the results obtained in the linear regression model identified a significant effect of the underresponsiveness/seek sensation and auditory filtering categories on the adaptive skills needed to participate in leisure activities or in environments such as home or school. Moreover, in the SSP, all items in the sensory sensitivity categories clearly represent hyperreactive sensory behaviors. In contrast to previous research, in our study [25–29], the results of the observed multiple regression models of these sensory categories did not show significant effects on adaptive abilities. Interestingly, in the resulting regression models that considered the sensory categories of movement sensitivity and auditory filtering, the genetic defect has a greater influence on adaptive social interaction skills than sensory processing skills.

### Cluster analysis

To our knowledge, this is the first study to attempt to explore phenotypic and genetic heterogeneity by considering the sensory characteristics and adaptive abilities of PMS patients. Of the five clusters, the cluster that grouped the participants with the largest mutation size (C5) was the one that showed the greatest sensory processing difficulties, compared to the rest of the group. In contrast, the cluster with the smallest mutation size (C2) showed better sensory processing results and higher adaptive skills. Although previous studies have considered that mutation size negatively and directly influences the severity of PMS phenotypes [48], the results obtained in this study seem to suggest the absence of marked differences regarding adaptive skills according to the size of the deletion. Nevertheless, our findings seem to support the fact that those patients with larger mutations have a clinical presentation characterized by greater adaptive difficulties and worse sensory processing skills.

The results obtained in this study facilitate the understanding of the impact that alterations in individual sensory processing and reactivity have on the adaptive skills of patients with PMS. In addition, the finding of a characteristic sensory profile could perhaps support the diagnosis of PMS pending the confirmation by genetic analysis. However, the phenotypic complexity of this syndrome makes longitudinal follow-up studies necessary to assess the evolution of skills, not only those related to sensory processing and adaptive behavior, but also those related to motor, communicative, and cognitive development. Future research should also include studies that evaluate the efficacy of current therapeutic interventions, as well as the design of new rehabilitation programs that consider the specific sensory needs and profiles of patients with PMS.

## Limitations

The sample size analyzed is relatively limited. However, PMS is a rare and under-diagnosed disease, and most published works have samples of the same or smaller size. This study relied on assessment tools that were self-administered by the primary caregiver, due to the patients’ degree of disability and the geographical distribution of the families in the study. These tools are validated, and their application is considered appropriate. However, the administration of tests or developmental batteries that can be administered directly, such as the Merrill-Palmer-Revised [49], Bayley Scales of Infant and Toddler Development [50], and Wechsler Preschool and Primary Scale of Intelligence [51], as well as tests for the evaluation of communication skills may be recommended and useful to define a more complete phenotypic profile of this population.

## Conclusion

The results of this study confirm the presence of an atypical sensory profile in patients with PMS. Moreover, they support a correlation between the dysfunction in sensory processing and limitations in adaptive skills affecting the daily life of PMS patients. Further studies are needed to assess whether the identification of specific sensory profiles could improve the diagnostic approach or therapeutic management of patients affected by this disease.

## Supplementary Information

Below is the link to the electronic supplementary material.

Supplementary file1 (DOCX 1532 KB)

## Data Availability

The data are available on request from the corresponding author. The data are not publicly available due to privacy aspects.

## Abbreviations

ABAS-II: Adaptive Behavior Assessment System II
ASD: autism spectrum disorder
CGH: comparative genomic hybridization
SSP-S: Short Sensory Profile-Spanish
PMS: Phelan-McDermid Syndrome
