# Supplementary material for: Sensory processing and adaptive behavior in Phelan-McDermid syndrome: a cross-sectional study
Source: Eur J Pediatr. 2022 Jul 15;181(8):3141–52. doi: 10.1007/s00431-022-04564-y (PMC9352617; doi:10.1007/s00431-022-04564-y)
Supplement: Supplementary file 1 — Supplementary file1 (DOCX 1532 KB) [file 431_2022_4564_MOESM1_ESM.docx]

**Sensory processing and adaptive behavior in Phelan-McDermid syndrome: a cross-sectional study**

Sergio Serrada-Tejeda^1^, María-Luz Cuadrado^2-3^, Rosa Mª Martínez-Piédrola^1^, Nuria Máximo-Bocanegra^1^, Patricia Sánchez-Herrera-Baeza^1^, Lucía Rocío Camacho-Montaño^1^ & Marta Pérez-de-Heredia-Torres^1^

**Affiliations**

^1^Department of Physical Therapy, Occupational Therapy, Rehabilitation and Physical Medicine. Rey Juan Carlos University. Avenida de Atenas s/n. CP.28922, Alcorcón, Madrid, Spain.

^2^ Department of Neurology, Hospital Clínico San Carlos, Madrid, Spain

^3^ Department of Medicine, School of Medicine, Universidad Complutense, Madrid, Spain

**Supplementary material**

1. Supplementary Figure 1. Flow diagram
2. Supplementary Figures 2-22. Short Sensory Profile and ABAS-II histograms
3. Supplementary Tables 1 and 2. Spearman correlation coefficients (r_s_) between the Short Sensory Profile scores and the ABAS-II adaptive skills and domains
4. Supplementary Scatterplot matrix 1. Significant correlations between SSP-S and ABAS-II skills
5. Supplementary Scatterplot matrix 2. Significant correlations between SSP-S and ABAS-II domains
6. Supplementary Tables 3-7. Multiple linear regression model of sensory categories on the ABAS-II
7. Supplementary Tables 8-9. Multiple linear regression model of SSP_total score_ on the ABAS-II

**Supplementary Figure 1.** Flow diagram.

69 families of the Spanish Association of Phelan McDermid

Analyzed sample

(n=51)

**ANALYSIS**

SSP-S and ABAS-II completion

(n=51)

Declined to participate (n= 14)

Missing values (n=0)

**ASSESSMENT**

Families that signed and accepted the informed consent form

(n=55)

Primary caregivers who did not complete the questionnaires (n= 4 )

**SELECTION**

**Histograms: Short Sensory Profile**

**Supplementary figure 2.** Tactile sensitivity histogram

**Supplementary figure 3.** Taste / smell sensitivity histogram


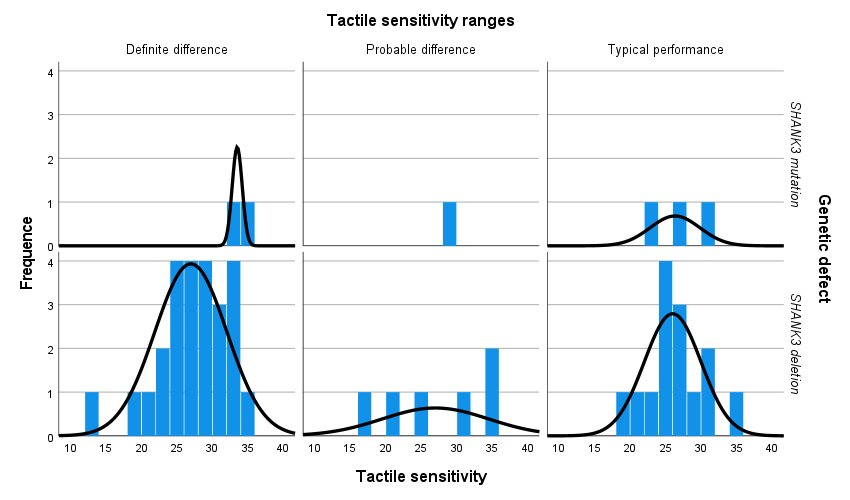

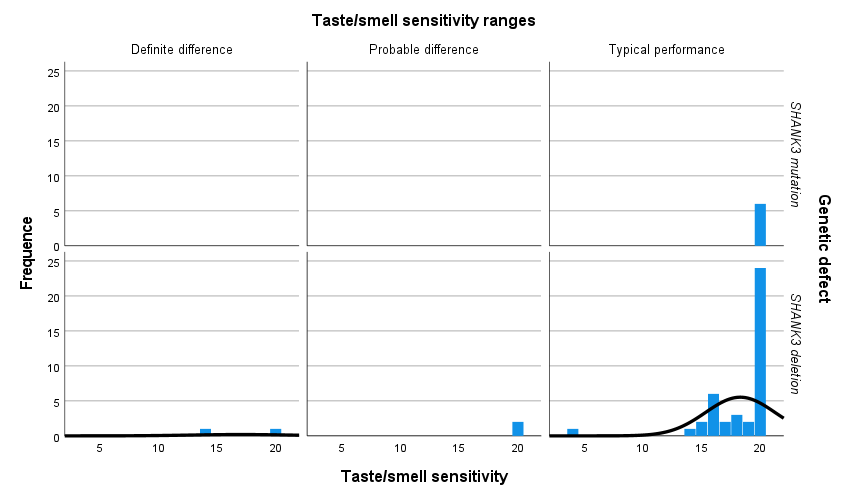


**Supplementary figure 5.** Underresponsive / seeks sensation histogram

**Supplementary figure 4.** Movement sensitivityhistogram


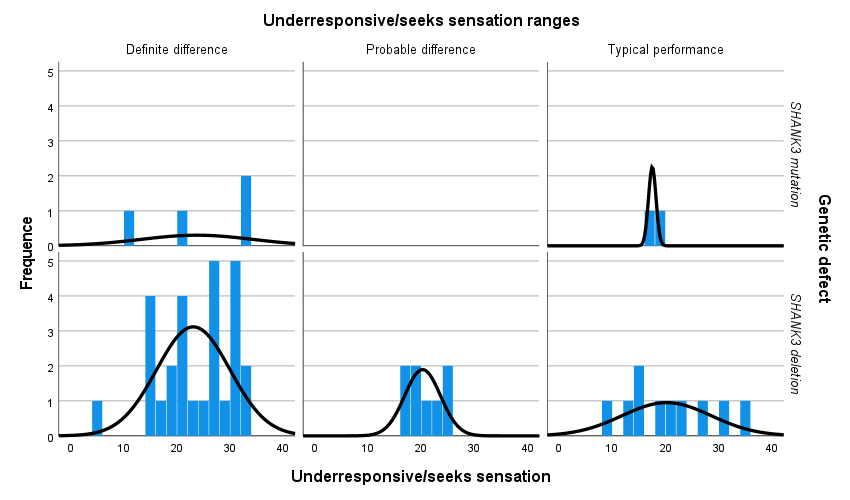

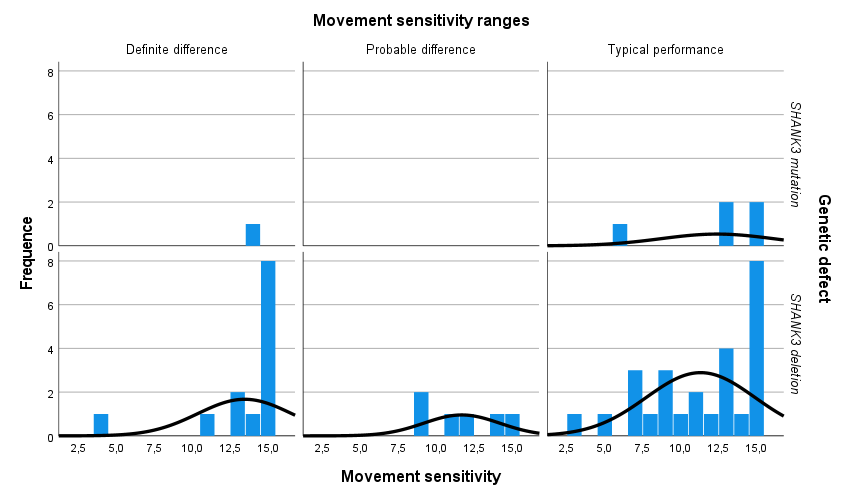


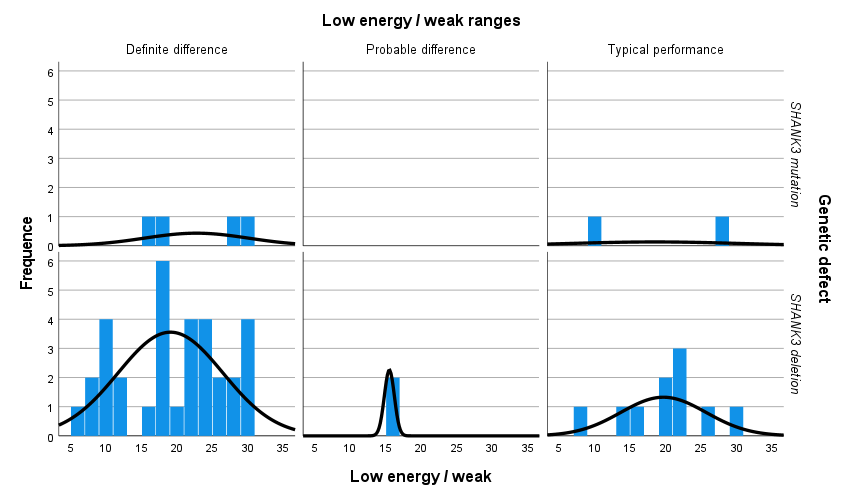

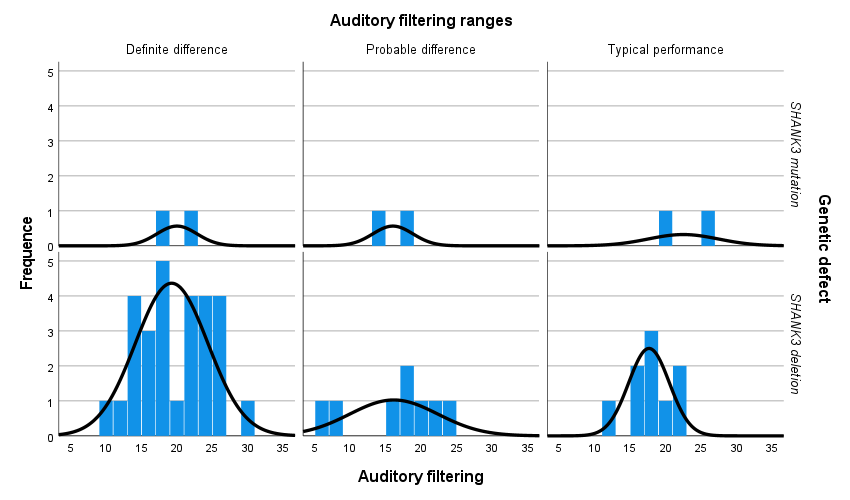


**Supplementary figure 6.** Auditory filtering histogram

**Supplementary figure 7**. Low energy /weak histogram

**Supplementary figure 9.** Short Sensory Profile Total Score histogram

**Supplementary figure 8.** Visual / auditory sensitivity histogram


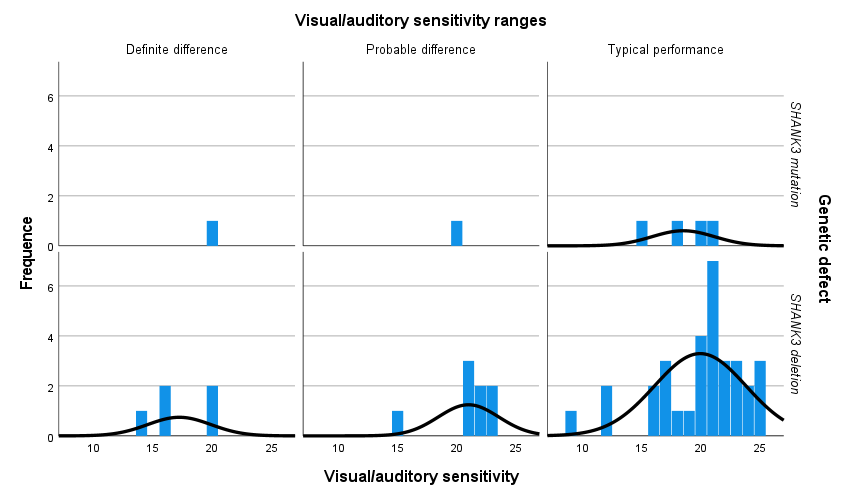

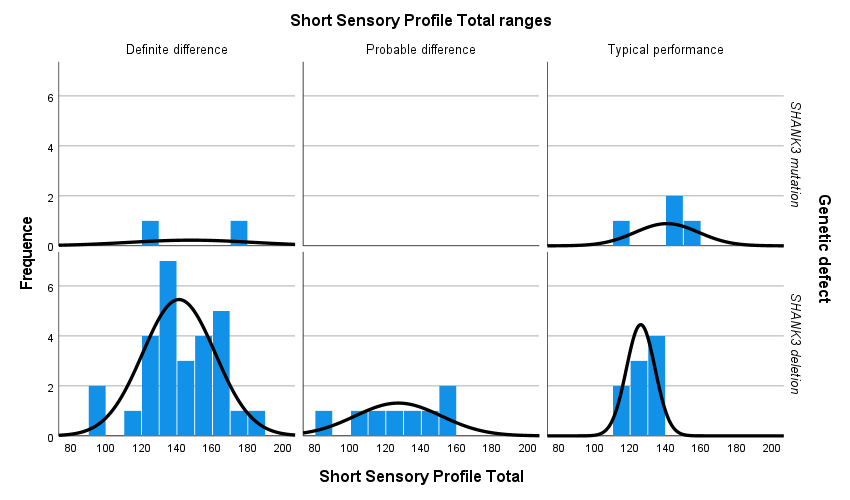


**Histograms: ABAS-II adaptive skills**

**Supplementary figure 10.** Communication adaptive skills histogram

**Supplementary figure 11.** Functional academic adaptive skills histogram


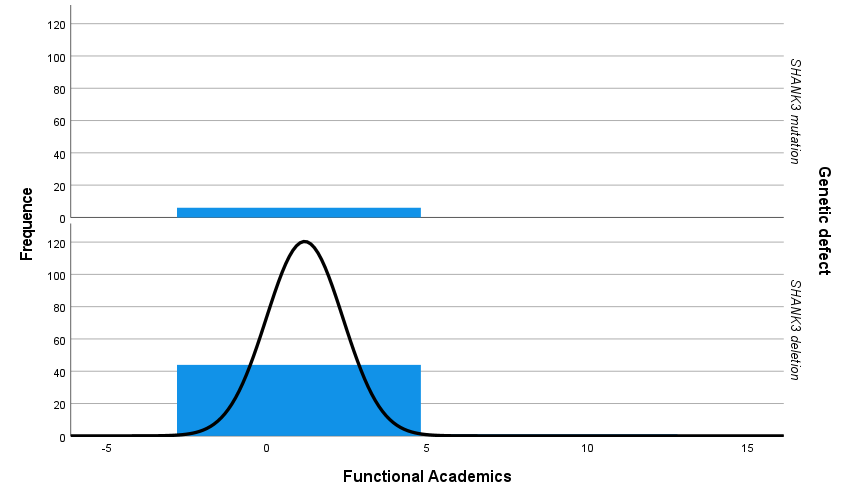

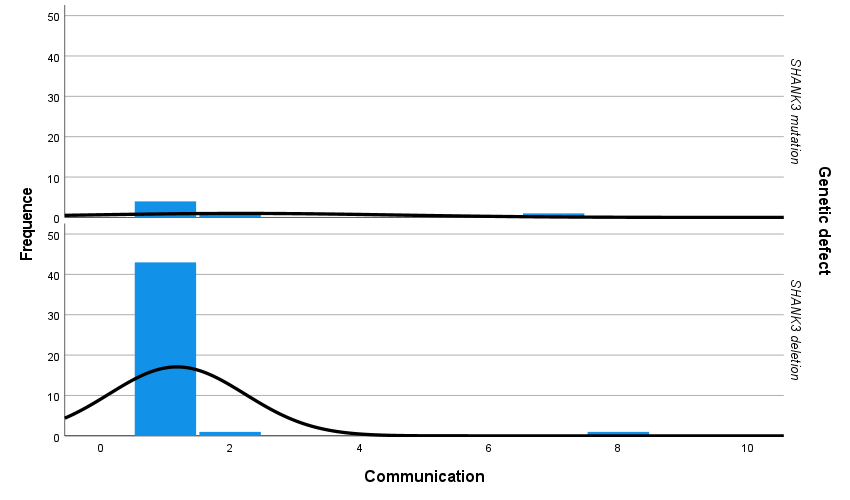


**Supplementary figure 13.** Leisure adaptive skills histogram

**Supplementary figure 12.** Self-Direction skills histogram


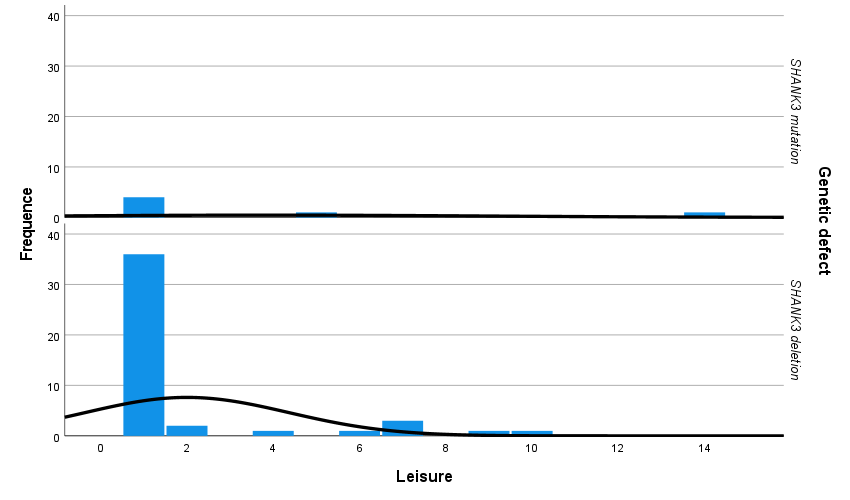

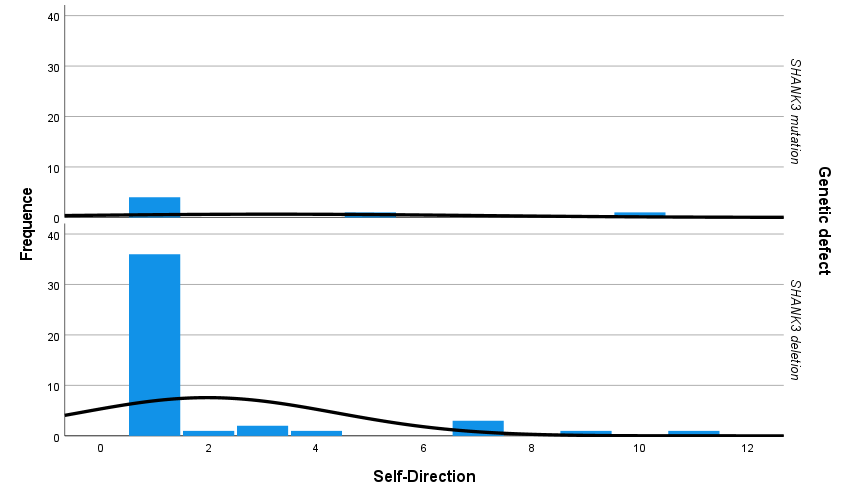


**Supplementary figure 15.** Community Use adaptive skills histogram

**Supplementary figure 14.** Social interaction adaptive skills histogram


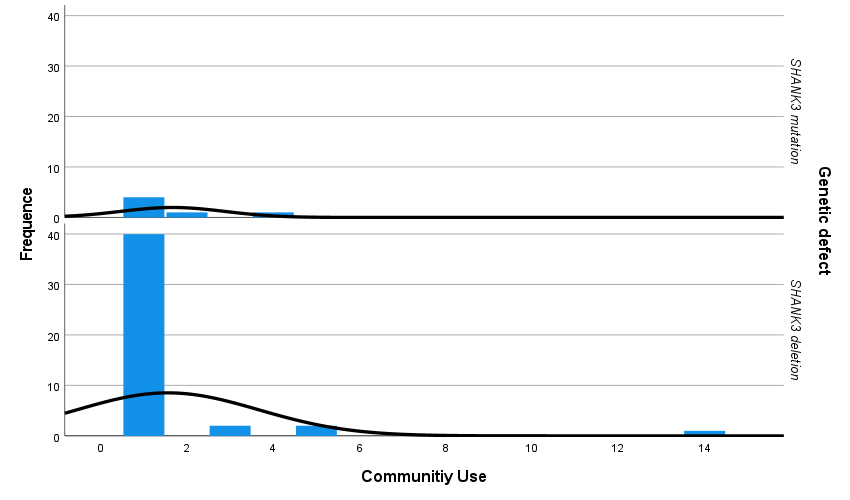

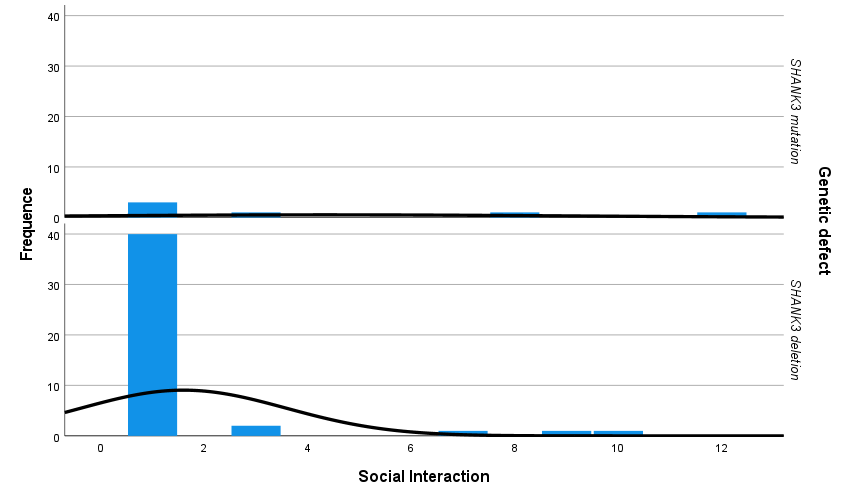


**Supplementary figure 17.** Health and Safety adaptive skills histogram

**Supplementary figure 16.** Home / School Living adaptive skills histogram


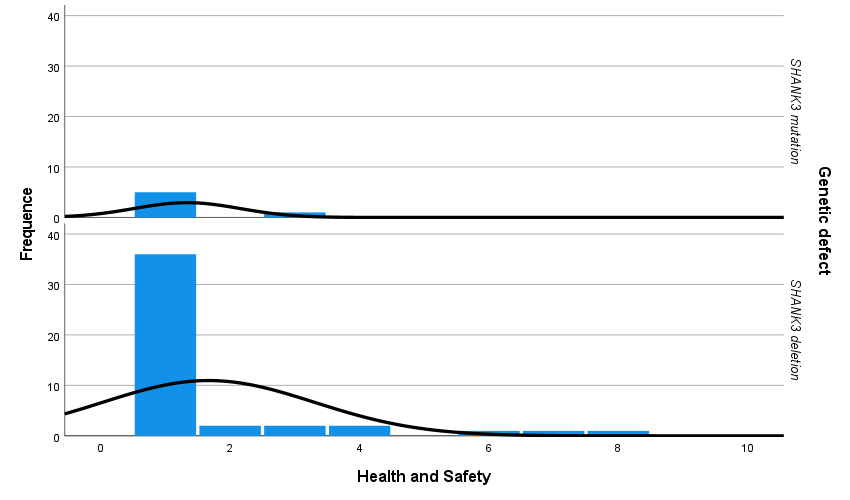

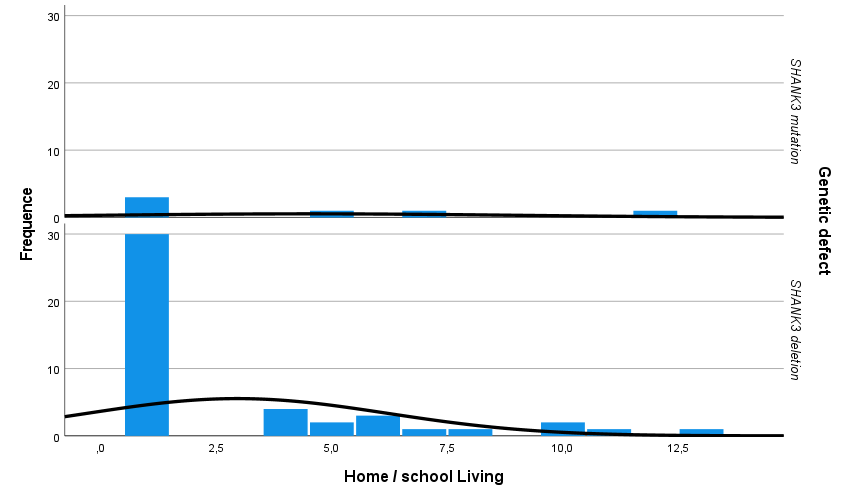


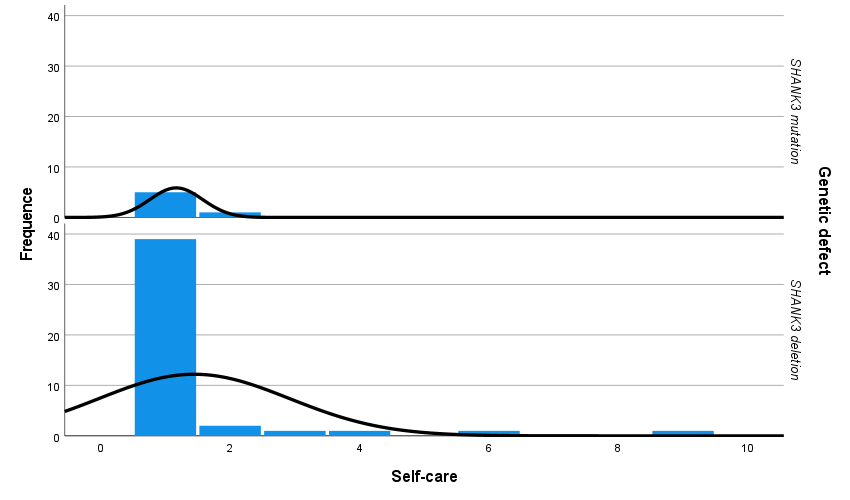


**Supplementary figure 18.** Health and Safety adaptive skills histogram

**ABAS-II Adaptive Domains**


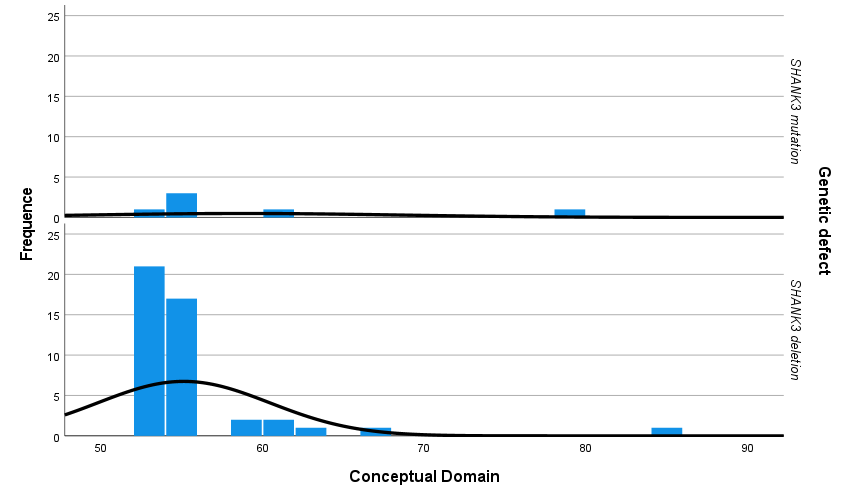


**Supplementary figure 20.** Social Domain histogram

**Supplementary figure 19.** Conceptual Domain histogram


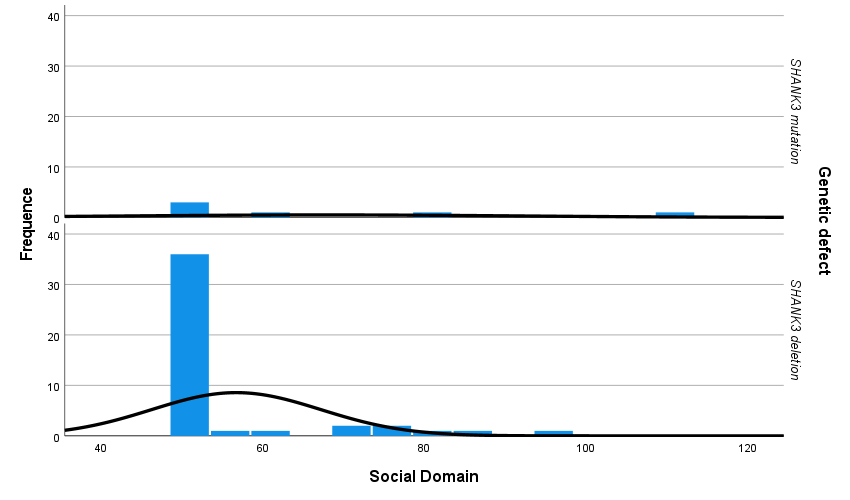


**Supplementary figure 22.** General Adaptive Composite histogram

**Supplementary figure 21.** Practical Domain histogram


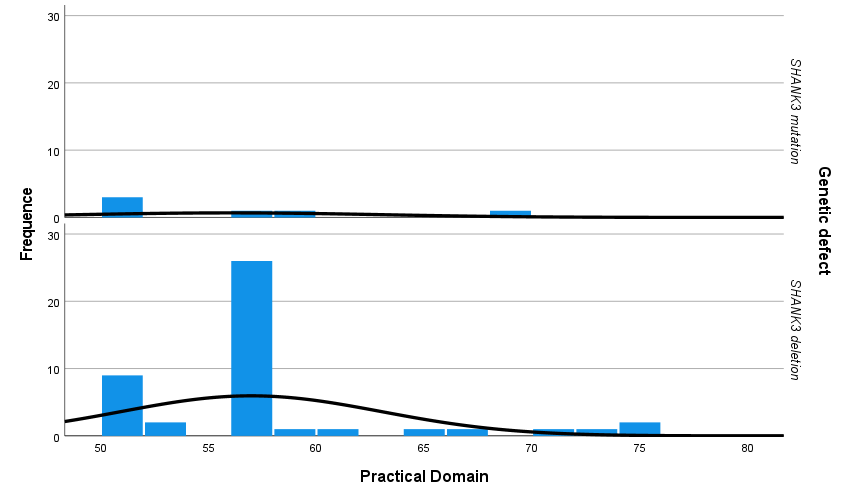


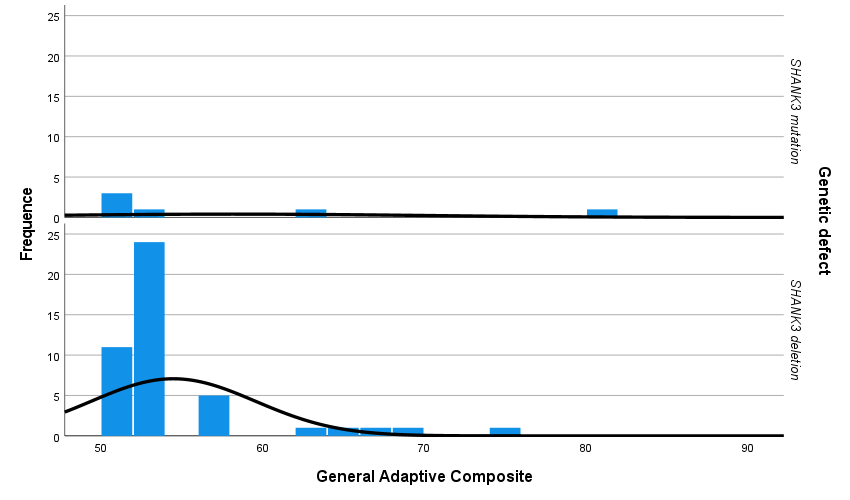


Positive and statistically significant correlations were found between the auditory filtering section of the SSP-S and the adaptive skills of self-direction (r_s_=0.405, p=0.003), leisure and social interaction (r_s_=0.435, p=0.001; r_s_=0.306, p=0.029), home or school living (r_s_=0.343, p=0.014), health and safety (r_s_=0.321, p=0.022) and self-care (r_s_=0.346, p=0.013). Statistically significant correlations were also observed between the underresponsive/seeks sensation section of the SSP-S and the adaptive behavior variables self-direction (r_s_=0.325, p=0.020), leisure (r_s_=0.295; p=0.036), home or school living (r_s_=0.381, p=0.006) and health and safety (r_s_=0.464, p=0.001). Similarly, statistically significant correlations were identified between movement sensitivity and social interaction skills (r_s_=0.276; p=0.050) and community use (r_s_=0.326; p=0.020). Finally, significant associations were found between SSP-S total score and the adaptive skills of self-direction (r_s_ =0.415, p=0.003), leisure and social interaction skills (r_s_=0.432, p=0.002; r_s_ 0.345, p=0.013), community use (r_s_=0.15; p= 0.024), home or school living (r_s_=0.453; p=0.001), health and safety (r_s_=0.368, p=0.008) and self-care (r_s_=0.314, p=0.025). Because scalar motor skill scores showed no variability across patients and were associated with very low adaptive skill profiles, it was not possible to assess the degree of correlation with other variables.

Supplementary Table 1. Spearman correlation coefficients (r_s_) between the Short Sensory Profile scores and the ABAS-II skill areas.

| *ABAS-II*  *SSP-S* | Communication | Functional  Academics | Self-Direction | Leisure  skills | Social Interaction Skills | Community Use | Home or School Living | Health and Safety | Self-Care |
| --- | --- | --- | --- | --- | --- | --- | --- | --- | --- |
| Tactile sensitivity | 0.063 | -0.231 | 0.114 | 0.162 | 0.191 | 0.245 | 0.564 | 0.139 | 0.142 |
| Taste/smell sensitivity | 0.046 | -0.197 | 0.105 | 0.142 | 0.106 | 0.172 | 0.119 | 0.068 | 0.061 |
| Movement sensitivity | 0.270 | 0.158 | 0.199 | 0.244 | **0.276*** | **0.326*** | 0.202 | 0.085 | 0.271 |
| Underresponsive/seeks sensation | -0.075 | -0.144 | **0.325*** | **0.295*** | 0.244 | 0.168 | **0.381**** | **0.464**** | 0.237 |
| Auditory filtering | 0.116 | -0.207 | **0.405**** | **0.435**** | **0.306*** | 0.176 | **0.343*** | **0.321*** | **0.346*** |
| Low energy/weak | -0.109 | -0.192 | 0.226 | 0.219 | 0.157 | 0.131 | 0.210 | 0.169 | 0.070 |
| Visual/auditory sensitivity | -0.118 | -0.228 | 0.084 | 0.166 | 0.098 | 0.081 | 0.107 | 0.022 | 0.068 |
| SSP-S Total Score | 0.059 | -0.231 | **0.415**** | **0.432**** | **0.345*** | **0.315*** | **0.453**** | **0.368**** | **0.314*** |

| SSP-S: Short Sensory Profile – Spanish; ABAS-II: Adaptive Behavior Assessment Questionnaire  ** p<0.01; * p<0.05 |
| --- |
|  |

There were positive and statistically significant correlations between SSP-S scores and the conceptual (rs=0.317; p=0.024), social (rs=0.419, p=0.002), practical (rs=0.453, p=0.001) and GAC index (rs=0.499, p=0.000) of the ABAS-II, whereby higher SSP-S scores were related to better skills and higher adaptive performance.

Supplementary Table 2. Spearman correlation coefficients (r_s_) between the Short Sensory Profile scores and the ABAS-II domains.

| *ABAS-II domain*  *SSP-S* | Conceptual | Social | Practical | GAC |
| --- | --- | --- | --- | --- |
| Tactile sensitivity | -0.014 | 0.237 | **0.316*** | **0.306*** |
| Taste/smell sensitivity | 0.217 | 0.037 | 0.053 | 0.103 |
| Movement sensitivity | 0.102 | **0.372**** | **0.365**** | **0.322*** |
| Underresponsive/seeks sensation | **0.283*** | 0.162 | 0.198 | 0.249 |
| Auditory filtering | **0.378**** | **0.354*** | 0.243 | **0.312*** |
| Low energy/weak | 0.141 | 0.254 | 0.234 | **0.296*** |
| Visual/auditory sensitivity | -0.066 | **0.290*** | **0.312*** | **0.316*** |
| Sensory Profile Total Score | **0.317*** | **0.419**** | **0.453**** | **0.499**** |

SSP-S: Short Sensory Profile – Spanish; ABAS-II: Adaptive Behavior Assessment Questionnaire; GAC: General adaptive composite

** p<0.01; * p<0.05


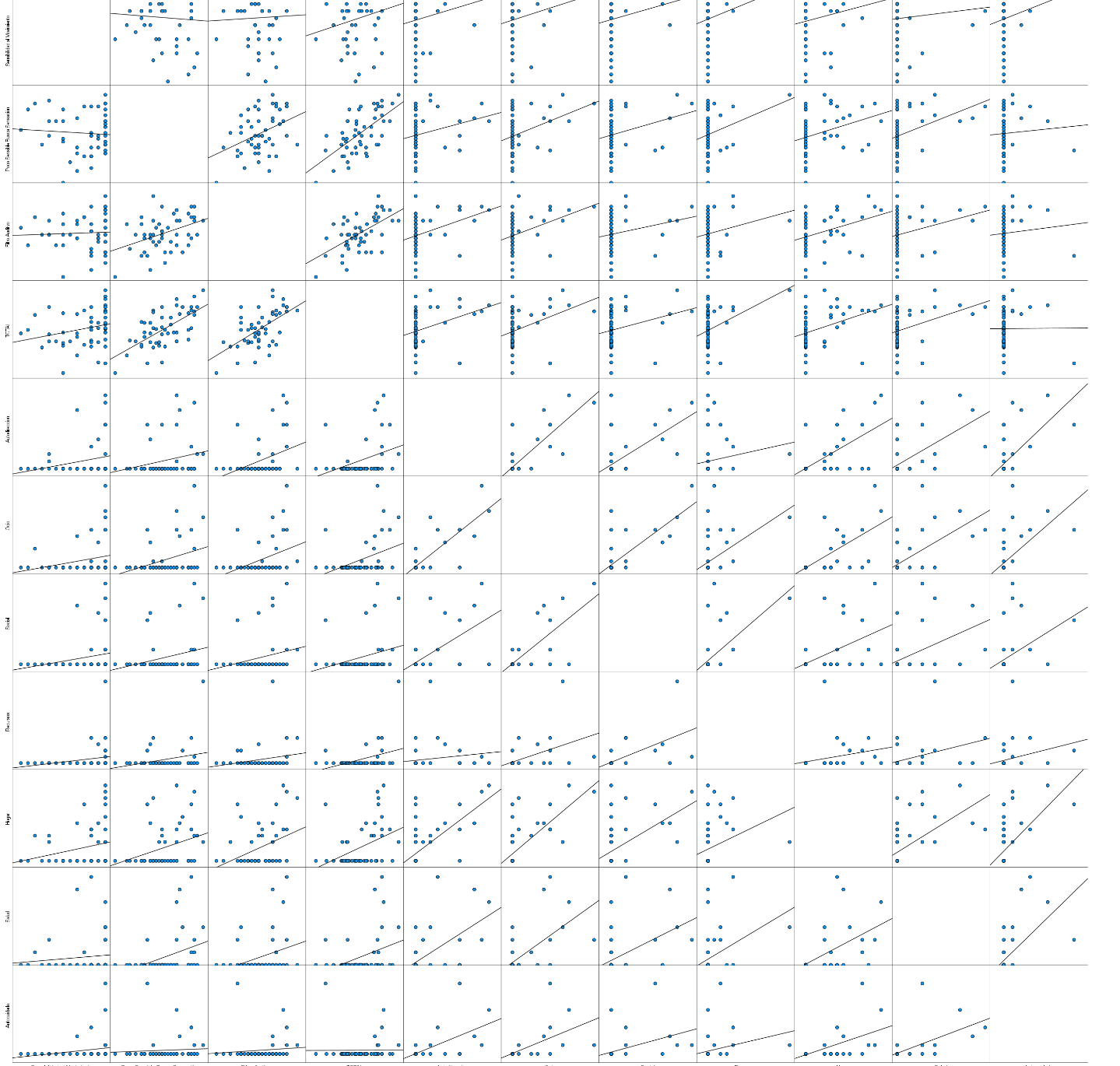


Supplementary Scatterplot matrix 1. Significant correlations between SSP-S and ABAS-II skills.

Movement Sensitivity

Underresponsive / seeks sensation

Auditory filtering

Auditory filtering

Underresponsive / seek sensation

Self-Care

Health and Safety

Home or

School Living

Community Use

Social

interaction skills

Leisure

Self-direction

SSP-S Total

Self-Care

Health and Safety

Home or

School Living

Community Use

Social

interaction skills

Leisure

Self-direction

SSP-S Total

Movement Sensitivity


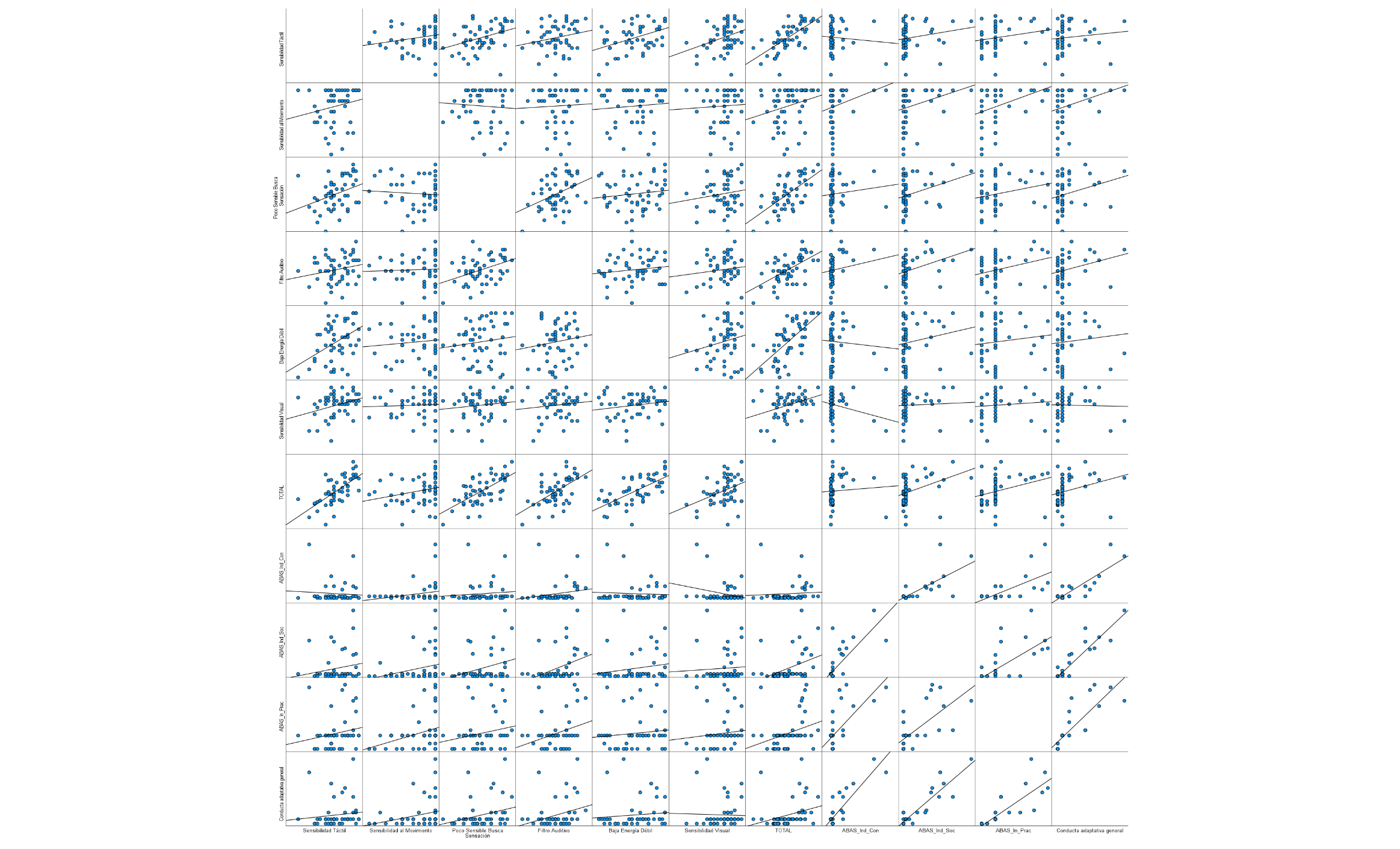


Supplementary Scatterplot matrix 2. Significant correlations between SSP-S and ABAS-II domains.

Tactile sensitivity

Practical Domain

Underresponsive / seek sensation

Movement sensitivity

Underresponsive / seek sensation

Tactile sensitivity

Auditory filtering

Low energy / weak

Visual/auditory

sensitivity

Visual/auditory sensitivity

SSP-S Total

Conceptual Domain

Social Domain

Practical Domain

GAC Index

Social Domain

CAG Index

Conceptual Domain

SSP-S Total

Low energy / weak

Auditory filtering

Movement sensitivity

Supplementary Table 3. Multiple linear regression model. Effect of *Tactile sensitivity* category, age and genetic defect on ABAS-II adaptive skills and domains

|  | Practical | | |  | GAC | | |
| --- | --- | --- | --- | --- | --- | --- | --- |
|  | **β (ET)** | ***t*** | ***p*-value** |  | **β (ET)** | ***t*** | **p-value** |
| Age | -0.25 (0.11) | -2.285 | **0.027** |  | -0.17 (0.11) | -1.491 | 0.143 |
| SHANK3_deletion_  vs  SHANK3_mutation_ | 1.37 (2.56) | 0.535 | 0.595 |  | -3.57 (2.66) | -1.342 | 0.186 |
| Tactile sensitivity | 0.19 (0.17) | 1.159 | 0.252 |  | 0.06 (0.17) | 0.362 | 0.719 |
|  |  |  |  |  |  |  |  |
| R^2^ (%) | 13.9 % | | |  | 8.7 % | | |
| Model | *F* (3,47) = 2.52, *p* > 0.05 | | |  | *F* (3,47) = 1.50, *p* > 0.05 | | |

Supplementary Table 4. Multiple linear regression model. Effect of *Movement sensitivity* category, age and genetic defect on ABAS-II adaptive skills and domains

|  | Social Interaction | | |  | Community Use | | |  | Social Domain | | | Practical Domain | | | |
| --- | --- | --- | --- | --- | --- | --- | --- | --- | --- | --- | --- | --- | --- | --- | --- |
|  | **β (ET)** | ***t*** | ***p*-value** |  | **β (ET)** | ***t*** | **p-value** |  | **β (ET)** | ***t*** | ***p*-value** |  | **β (ET)** | ***t*** | ***p*-value** |
| Age | 0.02 (0.05) | 0.510 | 0.613 |  | 0.02 (0.04) | 0.444 | 0.659 |  | -0.23 (0.29) | -0.077 | 0.939 |  | -0.15 (0.13) | -1.155 | 0.254 |
| SHANK3_deletion_  vs  SHANK3_mutation_ | -2.59 (1.03) | -2.500 | **0.016** |  | 0.00 (0.88) | 0.005 | 0.996 |  | -10.26 (5.37) | -1.910 | 0.062 |  | 1.21 (2.53) | 0.478 | 0.635 |
| Movement sensitivity | 0.19 (0.12) | 1.501 | 0.140 |  | 0.16 (0.10) | 1.484 | 0.144 |  | 0.94 (0.65) | 1.440 | 0.156 |  | 0.40 (0.31) | 1.291 | 0.203 |
|  |  |  |  |  |  |  |  |  |  |  |  |  |  |  |  |
| R^2^ (%) | 16.8 % | | |  | 5.2 % | | |  | 14.1 % | | |  | 14.4 % | | |
| Model | ***F* (3,47) = 3.17, *p* < 0.05** | | |  | *F* (3,47) = 0.85, *p* > 0.05 | | |  | F (3,47) = 2.57, p > 0.05 | | |  | F (3,47) = 2.64, p > 0.05 | | |

|  | CAG | | |
| --- | --- | --- | --- |
|  | **β (ET)** | ***t*** | ***p*-value** |
| Age | -0.078 (0.14) | -0.548 | 0.586 |
| SHANK3_deletion_  vs  SHANK3_mutation_ | -3.45 (2.61) | -1.322 | 0.193 |
| Movement sensitivity | 0.35 (0.31) | -1.322 | 0.193 |
|  |  |  |  |
| R^2^ (%) | 10.9 % | | |
| Model | *F* (3,47) = 1.91, *p* > 0.05 | | |

Supplementary Table 5. Multiple linear regression model. Effect of *Underresponsive/seek sensation* category, age and genetic defect on ABAS-II adaptive skills and domains

|  | **Self-Direction** | | |  | **Leisure** | | |  | **Home of School living** | | | **Health and Safety** | | | |
| --- | --- | --- | --- | --- | --- | --- | --- | --- | --- | --- | --- | --- | --- | --- | --- |
|  | **β (ET)** | ***t*** | ***p*-value** |  | **β (ET)** | ***t*** | **p-value** |  | **β (ET)** | ***t*** | ***p*-value** |  | **β (ET)** | ***t*** | ***p*-value** |
| Age | -0.06 (0.04) | -1.467 | 0.150 |  | -0.09 (0.04) | .1.928 | 0.060 |  | -0.09 (0.06) | -1.551 | 0.128 |  | -0.03 (0.02) | -1.230 | 0.225 |
| SHANK3_deletion_  vs  SHANK3_mutation_ | -1.23 (1.06) | -1.147 | 0.253 |  | -1.90 (1.10) | -1.714 | 0.093 |  | -1.61 (1.38) | -1.169 | 0.248 |  | 0.30 (0.63) | 0.472 | 0.639 |
| Underresponsivenss  /seek sensation | 0.10 (0.05) | 2.076 | **0.043** |  | 0.16 (0.05) | 3.137 | **0.003** |  | 0.18 (0.06) | 2.729 | **0.009** |  | 0.09 (0.03) | 3.080 | **0.003** |
|  |  |  |  |  |  |  |  |  |  |  |  |  |  |  |  |
| **R^2^ (%)** | 12.4 % | | |  | 23.2 % | | |  | 17.1 % | | |  | 17.7 % | | |
| **Model** | *F* (3,47) = 2.12, *p* > 0.05 | | |  | ***F* (3,47) = 4.74, *p* < 0.05** | | |  | **F (3,47) = 3.22, p < 0.05** | | |  | **F (3,47) = 3.36, p < 0.05** | | |

|  | **Conceptual Domain** | | |
| --- | --- | --- | --- |
|  | **β (ET)** | ***t*** | ***p*-value** |
| Age | -0.12 (0.11) | -0.987 | 0.329 |
| SHANK3_deletion_  vs  SHANK3_mutation_ | -3.75 (2.58) | -1.453 | 0.153 |
| Underresponsivenss  /seek sensation | 0.11 (0.12) | 0.928 | 0.358 |
|  |  |  |  |
| **R^2^ (%)** | 7 % | | |
| **Model** | *F* (3,47) = 1.18, *p* > 0.05 | | |

Supplementary Table 6. Multiple linear regression model. Effect of *Auditory filtering* category, age and genetic defect on ABAS-II adaptive skills and domains

|  | Self-Direction | | |  | Leisure | | |  | Social Interaction | | | Home of School Living | | | |
| --- | --- | --- | --- | --- | --- | --- | --- | --- | --- | --- | --- | --- | --- | --- | --- |
|  | **β (ET)** | ***t*** | ***p*-value** |  | **β (ET)** | ***t*** | **p-value** |  | **β (ET)** | ***t*** | ***p*-value** |  | **β (ET)** | ***t*** | ***p*-value** |
| Age | -0.06 (0.04) | -1.365 | 0.179 |  | -0.07 (0.04) | -1.572 | 0.123 |  | -0.03 (0.04) | -0.681 | 0.499 |  | -0.07 (0.05) | -1.262 | 0.213 |
| SHANK3_deletion_  vs  SHANK3_mutation_ | 0.98 (1.02) | -0.952 | 0.346 |  | -1.59 (1.11) | -1.430 | 0.159 |  | -2.61 (1.03) | -2.533 | **0.015** |  | -1.27 (1.38) | -0.922 | 0.361 |
| Auditory filtering | 0.20 (0.06) | 2.914 | **0.005** |  | 0.22 (0.07) | 3.046 | **0.004** |  | 0.11 (0.06) | 1.595 | 0.117 |  | 0.25 (0.09) | 2.806 | **0.007** |
|  |  |  |  |  |  |  |  |  |  |  |  |  |  |  |  |
| R^2^ (%) | 19 % | | |  | 22.5 % | | |  | 17.3 % | | |  | 17.7 % | | |
| Model | ***F* (3,47) = 3.67, *p* < 0.05** | | |  | ***F* (3,47) = 4.54, *p* < 0.05** | | |  | **F (3,47) = 3.28, p < 0.05** | | |  | **F (3,47) = 3.37, p < 0.05** | | |

|  | Health and Safety | | |  | Self-Care | | |  | Conceptual Domain | | | Social Domain | | | |
| --- | --- | --- | --- | --- | --- | --- | --- | --- | --- | --- | --- | --- | --- | --- | --- |
|  | **β (ET)** | ***t*** | ***p*-value** |  | **β (ET)** | ***t*** | **p-value** |  | **β (ET)** | ***t*** | ***p*-value** |  | **β (ET)** | ***t*** | ***p*-value** |
| Age | -0.02 (0.02) | -0.791 | 0.433 |  | -0.03 (0.02) | -1.238 | 0.222 |  | -0.10 (0.11) | -0.131 | 0.353 |  | -0.34 (0.21) | -1.588 | 0.119 |
| SHANK3_deletion_  Vs  SHANK3_mutation_ | 0.44 (0.66) | 0.672 | 0.505 |  | 0.30 (0.61) | 0.502 | 0.618 |  | -3.47 (2.57) | -1.352 | 0.183 |  | -9.98 (5.03) | -1.963 | 0.056 |
| Auditory filtering | 0.10 (0.04) | 2.352 | **0.023** |  | 0.03 (0.04) | 0.800 | 0.428 |  | 0.25 (0.17) | 1.309 | 0.197 |  | 0.99 (0.33) | 2.967 | **0.005** |
|  |  |  |  |  |  |  |  |  |  |  |  |  |  |  |  |
| R^2^ (%) | 11.5 % | | |  | 4.5 % | | |  | 8.7 % | | |  | 24.5 % | | |
| Model | *F* (3,47) = 2.10, *p* > 0.05 | | |  | *F* (3,47) = 0.73, *p* < 0.05 | | |  | F (3,47) = 1.48, p < 0.05 | | |  | **F (3,47) = 5.07, p < 0.05** | | |

|  | CAG | | |
| --- | --- | --- | --- |
|  | **β (ET)** | ***t*** | ***p*-value** |
| Age | -0.19 (0.10) | -1.842 | 0.072 |
| SHANK3_deletion_  vs  SHANK3_mutation_ | -3.31 (2.51) | -1.318 | 0.194 |
| Auditory filtering | 0.37 (0.16) | 2.220 | **0.031** |
|  |  |  |  |
| R^2^ (%) | 17.2 % | | |
| Model | ***F* (3,47) = 3.24, *p* < 0.05** | | |

Supplementary Table 7. Multiple linear regression model. Effect of *Low energy/weak and Visual and Auditory sensitivity* categories, age and genetic defect on ABAS-II adaptive skills and domains

|  | GAC | | |  |
| --- | --- | --- | --- | --- |
|  | **β (ET)** | ***t*** | ***p*-value** |  |
| Age | -0.17 (0.11) | -1.504 | 0.139 |  |
| SHANK3_deletion_  vs  SHANK3_mutation_ | -3.60 (2.64) | -1.364 | 0.179 |  |
| Low Energy/weak | 0.05 (0.12) | 0.412 | 0.682 |  |
|  |  |  |  |  |
| R^2^ (%) | 8.8 % | | |  |
| Model | *F* (3,47) = 1.51, *p* > 0.05 | | |  |

|  | Social Domain | | |  | Practical Domain | | |  | CAG | | |
| --- | --- | --- | --- | --- | --- | --- | --- | --- | --- | --- | --- |
|  | **β (ET)** | ***t*** | ***p*-value** |  | **β (ET)** | ***t*** | **p-value** |  | **β (ET)** | ***t*** | ***p*-value** |
| Age | -0.27 (0.23) | -1.157 | 0.253 |  | -0.26 (0.11) | -2.390 | 0.021 |  | -0.17 (0.11) | -0.219 | 0.125 |
| SHANK3_deletion_  Vs  SHANK3_mutation_ | -11.17 (5.47) | -2.043 | **0.047** |  | 0.81 (2.57) | 0.317 | 0.752 |  | -3.68 (2.64) | -1.395 | 0.170 |
| Visual/auditory  sensitivity | 0.24 (0.51) | 0.474 | 0.638 |  | 0.11 (0.23) | 0.480 | 0.633 |  | -0.05 (0.24) | -0.204 | 0.839 |
|  |  |  |  |  |  |  |  |  |  |  |  |
| R^2^ (%) | 10.7 % | | |  | 11.8 % | | |  | 8.6 % | | |
| Model | *F* (3,47) = 1.88, *p* > 0.05 | | |  | *F* (3,47) = 2.10, *p* > 0.05 | | |  | F (3,47) = 1,46,  *p* > 0.05 | | |

Table 8. Multiple linear regression model. Effect of SSP_total score_, age and genetic defect on ABAS-II adaptive skills, adaptive domains and CAG

|  | Functional academics | | |  | Self-Direction | | |  | Leisure | | | Social Interaction | | | | |
| --- | --- | --- | --- | --- | --- | --- | --- | --- | --- | --- | --- | --- | --- | --- | --- | --- |
|  | **β (ET)** | ***t*** | ***p*-value** |  | **β (ET)** | ***t*** | **p-value** |  | **β (ET)** | ***t*** | ***p*-value** |  | **β (ET)** | ***t*** | ***p*-value** |  |
| Age | -0.15 (0.21) | -0.747 | 0.459 |  | -0.04 (0.04) | -0.955 | 0.344 |  | -0.05 (0.04) | -1.143 | 0.259 |  | -0.02 (0.04) | -0.455 | 0.652 |  |
| SHANK3_deletion_ vs SHANK3_mutation_ | 0.05 (0,48) | 0.120 | 0.905 |  | -0.88 (1.05) | -0.839 | 0.406 |  | -1.46 (1.13) | -1.286 | 0.205 |  | -2.51 (1.03) | -2.439 | **0.019** |  |
| SSP_total score_ | -0.01 (0.08) | -1.981 | 0.053 |  | 0.04 (0.01) | 2.436 | **0.019** |  | 0.05 (0.01) | 2.784 | **0.008** |  | 0.02 (0.01) | 1.758 | 0.085 |  |
|  |  |  |  |  |  |  |  |  |  |  |  |  |  |  |  |  |
| R^2^ (%) | 7,6% | | |  | 15.1% | | |  | 20.3% | | |  | 18.2% | | | |
| Model | *F* (3,47) = 1.20, *p* > 0.05 | | |  | ***F* (3,47) = 2.77, *p* < 0.05** | | |  | **F (3,47) = 3.99, p < 0.05** | | |  | **F (3,47) = 3.49, p < 0.05** | | | |
|  | | | | | | | | | | | | | | | | |

|  | Community Use | | |  | Home or School Living | | |  | Health and Safety | | | Self-Care | | | |
| --- | --- | --- | --- | --- | --- | --- | --- | --- | --- | --- | --- | --- | --- | --- | --- |
|  | **β (SE)** | ***t*** | ***p*-value** |  | **β (SE)** | ***t*** | **p-value** |  | **β (SE)** | ***t*** | ***p*-value** |  | **β (SE)** | ***t*** | ***p*-value** |
| Age | -0.18 (0.03) | -0.519 | 0.612 |  | -0.05 (0.05) | -0.873 | 0.387 |  | -0.01 (0.02) | -0.456 | 0.650 |  | -0.03 (0.02) | -1.154 | 0.254 |
| SHANK3_deletion_ vs SHANK3_mutation_ | 0.16 (0.84) | 0.189 | 0.851 |  | -1.08 (1.38) | -0.781 | 0.439 |  | 0.54 (0.65) | 0.838 | 0.406 |  | 0.27 (0.61) | 0.443 | 0.660 |
| SSP_total score_ | 0.03 (0.01) | 2.699 | **0.010** |  | 0.06 (0.02) | 2.835 | **0.007** |  | 0.02 (0.01) | 2.738 | **0.009** |  | 0.0 (0.1) | 0.042 | 0.967 |
|  |  |  |  |  |  |  |  |  |  |  |  |  |  |  |  |
| R^2^ (%) | 14.1 % | | |  | 18% | | |  | 14.7% | | |  | 3.2% | | |
| Model | F (3,47) = 2.56; p > 0.05 | | |  | **F (3,47) = 3.42; p < 0.05** | | |  | F (3,47) = 2.69; p > 0.05 | | |  | F (3,47) = 0.51; p > 0.05 | | |

Note: β: regression coefficient.SE: standard error; R^2^: adjusted coefficient of determination; SSP: Short Sensory Profile

Table 9. Multiple linear regression model: Effect of SSP, age and genetic defect on ABAS-II adaptive domain and GAC index.

|  | Conceptual Domain | | |  | Social Domain | | |  | Practical Domain | | | GAC | | | |
| --- | --- | --- | --- | --- | --- | --- | --- | --- | --- | --- | --- | --- | --- | --- | --- |
|  | **β (ET)** | ***t*** | ***p*-value** |  | **β (ET)** | ***t*** | **p-value** |  | **β (ET)** | ***t*** | ***p*-value** |  | **β (ET)** | ***t*** | ***p*-value** |
| Age | -0.88 (0.11) | -0.789 | 0.434 |  | -0.25 (0.21) | -1.170 | 0.248 |  | -0.25 (0.10) | -2.468 | **0.017** |  | -0.16 (0.10) | -1.528 | 0.133 |
| SHANK3_deletion_ vs SHANK3_mutation_ | -3.64 (2.62) | -1.388 | 0.172 |  | -9.28 (5.11) | -1.813 | 0.076 |  | 1.61 (2.44) | 0.659 | 0.513 |  | -3.14 (2.56) | -1.228 | 0.225 |
| SSP_total score_ | 0.01 (0.04) | 0.231 | 0.818 |  | 0.24 (0.08) | 2.739 | **0.009** |  | 0.09 (0.03) | 2.363 | **0.022** |  | 0.07 (0.04) | 1.851 | 0.070 |
|  |  | | |  |  | | |  |  | | |  |  |  |  |
| R^2^ (%) | 5.4% | | |  | 22.7% | | |  | 20.8% | | |  | 14.7% | | |
| Model | F (3,47) = 0.90, p >0.05 | | |  | **F (3,47) = 4.59, p< 0.05** | | |  | **F (3,47) = 4.11; p< 0,05** | | |  | F (3,47) = 2.70; p> 0.05 | | |

Note: β: regression coefficient.SE: standard error; R^2^: adjusted coefficient of determination; SSP: Short Sensory Profile; GAC: General adaptive composite
